# Complex interactions of lovastatin with 10 chemotherapeutic drugs: a rigorous evaluation of synergism and antagonism

**DOI:** 10.1186/s12885-021-07963-w

**Published:** 2021-04-06

**Authors:** Kaitlyn A. Khandelwal Gilman, Seungmin Han, Young-Wook Won, Charles W. Putnam

**Affiliations:** 1 Arizona Cancer Center, University of Arizona, Tucson, AZ USA; 2 Division of Cardiothoracic Surgery, Department of Surgery, College of Medicine-Tucson, University of Arizona, Tucson, AZ USA

**Keywords:** Statins, Chemotherapy, Drug interaction, Synergism, Antagonism, Loewe model, Combenefit, Dual drug assay, Tamoxifen

## Abstract

**Background:**

Evidence bearing on the role of statins in the prevention and treatment of cancer is confounded by the diversity of statins, chemotherapeutic agents and cancer types included in the numerous published studies; consequently, the adjunctive value of statins with chemotherapy remains uncertain.

**Methods:**

We assayed lovastatin in combination with each of ten commonly prescribed chemotherapy drugs in highly reproducible in vitro assays, using a neutral cellular substrate, *Saccharomyces cerevisiae.* Cell density (OD_600_) data were analyzed for synergism and antagonism using the Loewe additivity model implemented with the Combenefit software.

**Results:**

Four of the ten chemotherapy drugs – tamoxifen, doxorubicin, methotrexate and rapamycin – exhibited net synergism with lovastatin. The remaining six agents (5-fluorouracil, gemcitabine, epothilone, cisplatin, cyclophosphamide and etoposide) compiled neutral or antagonistic scores. Distinctive patterns of synergism and antagonism, often coexisting within the same concentration space, were documented with the various combinations, including those with net synergism scores. Two drug pairs, lovastatin combined with tamoxifen or cisplatin, were also assayed in human cell lines as proof of principle.

**Conclusions:**

The synergistic interactions of tamoxifen, doxorubicin, methotrexate and rapamycin with lovastatin – because they suggest the possibility of clinical utility - merit further exploration and validation in cell lines and animal models. No less importantly, strong antagonistic interactions between certain agents and lovastatin argue for a cautious, data-driven approach before adding a statin to any chemotherapeutic regimen. We also urge awareness of adventitious statin usage by patients entering cancer treatment protocols.

**Supplementary Information:**

The online version contains supplementary material available at 10.1186/s12885-021-07963-w.

## Background

From antiquity^1^ [1] to the present day cancer has plagued humanity; in 2018 cancer claimed an estimated 9.6 million lives, one in six deaths worldwide [2]. Despite the many consequential improvements in cancer treatment, there remains a clinical imperative to identify novel therapies with improved efficacy and diminished toxicity. In this context, statins [3-hydroxy-3-methylglutaryl-CoA (HMG-CoA) reductase inhibitors], the most commonly prescribed class of pharmaceuticals worldwide, have engendered promise and drawn scrutiny.

In 1971 Akira Endo isolated the first statin progenitor, citrinin [3]. Soon it was shown to be an inhibitor of HMG-CoA reductase [4], the rate limiting step in the mevalonate pathway. Although citrinin proved to be nephrotoxic, in 1976 the Endo laboratory [5, 6] and a British group [7] independently isolated ML-236B, also called compactin. Eleven years later, the FDA approved the first commercial statin: naturally derived lovastatin. Subsequently six statins, including two semi-synthetic and four synthetic formulations, have entered the marketplace and represent primary therapy for the prevention of cardiovascular disease. Today, an estimated 30 million people worldwide take statins.

Within a few years of the introduction of statins, however, concerns regarding their safety emerged, notably an associated increase in non-cardiac mortality [8]. Animal studies suggested that they might be carcinogenic: when given statins at doses equivalent to those commonly prescribed in humans, rats developed lymphomas and carcinomas of the liver, stomach, lung, and thyroid [9]. Consequently, large randomized clinical trials were undertaken to evaluate not only the efficacy of statins but also any associated risk of cancer. Ironically, by demonstrating reduced incidences of colorectal carcinoma, prostate cancer and melanoma [10], these studies were the first to indicate that statins might prevent cancer. To date, preclinical and clinical data suggest chemopreventive effects of statins against a variety of cancers including those of the breast [11], colon [12], lung [13], liver [14], pancreas [15], and prostate [16].

Several lines of evidence have suggested that statins might also have value in the treatment of cancer: statins modulate the mevalonate pathway [17], which ultimately modifies the posttranslational processing of proteins involved in cell cycle control; cancer cells exhibit increased synthesis, receptor mediated uptake, and degradation of cholesterol (reviewed in [18]); and, disrupted cholesterol homeostasis has been demonstrated in various tumor models (see, for example, [19]). In 1998, Matar, et al. [20], published a landmark study: a short course of lovastatin in rats inhibited primary fibrosarcoma growth and diminished the size and number of experimentally induced lung metastases. Subsequently, numerous publications have supported the notion that statins exert anticancer activity through mevalonate-dependent and -independent mechanisms, as recently reviewed [17, 21].

Disappointingly, statins *alone* have not proven effective as anticancer therapy; however, there is evidence that statins might potentiate the effects of anti-cancer drugs [22]. A recent systematic review and meta-analysis by Mei et al. [23] (which included 95 studies, 1,111,407 patients and more than 18 cancer types) compared statin users to individuals not taking statins. The patients receiving statins in conjunction with chemotherapy experienced a 30% reduction in all-cause mortality, a 40% reduction in cancer-specific mortality, and prolonged progression-free, recurrence-free, and disease-free survivals. Yet soon after the publication of Mei’s analysis, Farooqui et al. [24] published a systematic review and meta-analysis of 10 randomized controlled studies involving 1881 individuals with stage 3 or 4 cancers, in which statin use did not improve progression-free or overall survival.

Reaching a sound assessment from clinical trials of the value of statins as adjuncts to conventional chemotherapy is confounded by the numbers of different drugs – both statins and chemotherapeutic agents – featured in the various investigations, and the multiplicity of cancer types treated. We reasoned that the possible therapeutic benefits of statins in the context of chemotherapy are unlikely the global consequence of statin administration but instead are specific to the interacting drug combinations. Therefore, we chose to investigate a subset of statin – chemotherapeutic drug interactions by rigorously assaying a single statin, lovastatin (which has similar dissociation constants with HMG Co-A reductase from budding yeast and mammalian sources [25]), in combination with each of ten FDA-approved chemotherapeutic agents having a variety of mechanisms of action.

Because our objective was to compare the ten drug pairings with each other, it was important to use a neutral cellular substrate, rather than a particular human cancer cell line which because of its inherent cellular origin and genetic mutations might favor or disadvantage a particular drug pair. We therefore chose as the cellular substrate the exceptionally well-studied model organism, *Saccharomyces cerevisiae* [26]. There is ample precedent for utilizing budding yeast in research related to cancer and its therapies [26–28]. Many of its genes have human orthologs [29] and many cell signaling pathways now recognized as critical to oncogenesis were first identified and/or extensively studied in yeast. Importantly, *S. cerevisiae* has been utilized in numerous pharmacologic studies and high throughput screens [30–40], including ones specifically focused on anticancer drug research (reviewed in [41]). In anticipation of future studies of the genetic basis of interactions of statin-drug combinations, we created a balanced pool of heterozygous deletion strains (marked by DNA bar codes) of *S. cerevisiae* essential genes. Barcoded pools [42] were employed in the three largest yeast chemogenomic screens [32, 34, 36] which are collated in the NetwoRx data base [33]. (The pools also provide a resource for investigating genetically driven resistance to drug treatments [43, 44].) We configured a 96-well microplate assay compatible with the *Combenefit* dual-drug interaction software [45]; cell concentration data, read spectrophotometrically, were submitted to rigorous statistical analysis for synergistic or antagonistic interactions, calculated according to the Loewe additivity model [46] which is part of the *Combenefit* package.

Our data demonstrate that combining lovastatin with conventional chemotherapeutic agents results in drastically different interactions, ranging from strong synergism to profound antagonism, sometimes within the same concentration space. Of the ten chemotherapeutic drugs, four (tamoxifen, doxorubicin, methotrexate, and rapamycin) exhibited net synergism with lovastatin; two drugs (gemcitabine and 5-fluorouracil) had neutral scores; and four (epothilone, cisplatin, cyclophosphamide, and etoposide) displayed net antagonism. As proof of principle, two of the drug combinations, tamoxifen/lovastatin and cisplatin/lovastatin, were further evaluated in human cancer cell lines. The results in cell lines were generally accordant with the data obtained with *S. cerevisiae* but with variations in the patterns of synergism and antagonism between individual cell lines, even of the same cancer type.

## Methods

### *Saccharomyces cerevisiae* heterozygous deletion pool

The complete collection of *S. cerevisiae* heterozygous diploid essential gene deletion strains was obtained from ThermoScientific. A balanced pool was created, aliquoted and frozen (see Additional file 1: Methods). For each set of assays, 10 ml of synthetic complete media with 2% dextrose (SCD) was inoculated with 10 *μ*l of pooled cells and incubated overnight in a shaking water bath at 29 °C. Before using in assays, the cells were diluted with SCD to an OD_600_ reading of 0.7–0.85.

### Chemotherapeutic and statin drugs

The following drugs were obtained from Cayman Chemical (Ann Arbor, MI, USA): cisplatin (cis-Diamminedichloroplatinum), cyclophosphamide (hydrate), epothilone B, etoposide, 5-fluorouracil, lovastatin (lovastatin hydroxy acid, sodium salt), rapamycin, and tamoxifen. Compounds obtained from Millipore Sigma included: doxorubicin hydrochloride, gemcitabine hydrochloride, and methotrexate.

### *Saccharomyces cerevisiae* dose response curves

In order to determine suitable concentration ranges for the dual drug dilution assays, dose response curves were compiled for each of the 11 drugs, using a range of concentrations based upon experimental data in *S. cerevisiae* from the NetwoRx data base [33] and/or published reports [34, 36, 47, 48] The methodology is described in detail in Additional file 1: Methods.

### *Saccharomyces cerevisiae* dual drug dilution assay

Dual drug assays were performed in 96-well tissue culture plates using a uniform format compatible with the input requirements of the Combenefit software package (see later). In each assay, lovastatin was paired with a single chemotherapeutic drug. A schematic diagram of the plate configuration is shown in Additional file 2: Fig. S1, and the methodology for the assay is described in detail in Additional file 1: Methods. The concentration ranges for each drug tested are given in Table 1. Crosswise, serial 70% dilutions create a matrix of 49 unique dual-drug combinations; the plates also include individual drug dose responses along with positive (cells only) and negative (SCD only) control wells. Plates are sealed and incubated with shaking at 29 °C. Cell densities are read using a plate reader (OD_600_), after two and 3 days of incubation. Each experiment was conducted a minimum of 3 times; the numbers of biologic replicates are given in the figure legends.

**Table 1:** Concentration ranges of the ten chemotherapeutic drugs and lovastatin in the *S. cerevisiae* assays

Drug	Principal mechanism of action	Concentration Range (μM)
Cisplatin	DNA crosslinking	12.4–105
Cyclophosphamide	Alkylating agent which binds to DNA	49.4–420
Doxorubicin	Intercalates DNA, blocking replication	24.7–210
Epothilone	Binds to microtubules, blocking cell division	1.1–9.3
Etoposide	Topoisomerase II inhibitor	0.165–1.4
5-Fluorouracil	Antimetabolite: blocks thymidylate synthetase	0.92–8.07
Gemcitabine	Antimetabolite: incorporated into DNA in place of dCTP	14.4–123
Methotrexate	Antimetabolite: binds to dihydrofolate reductase	8.24–70.0
Rapamycin	mTOR inhibitor	0.33–2.80
Tamoxifen	Selective estrogen receptor modulator (SERM)	24.7–210
Lovastatin	Competitive inhibitor of HMG-CoA reductase	12.7–108

### Assays in human cell lines

Crosswise, dual-dilution format assays were also performed using human cancer cell lines as substrate. The cell lines, obtained from ATCC, included: A549 (adenocarcinoma, lung), HCC827 (adenocarcinoma, lung), HT-29 (colorectal adenocarcinoma), MCF7 (metastatic adenocarcinoma, breast, estrogen, progesterone and growth factor receptor expressing), MDA-MB-231 (metastatic adenocarcinoma, breast, triple negative for receptor expression), and SK-BR-3 (metastatic adenocarcinoma, breast, HER2 overexpressing). The cells were cultured according to the corresponding ATCC protocols.

The dual drug crosswise dilution matrix assays were performed in a similar format as the *S. cerevisiae* experiments and likewise included a plate blank, individual drug dose responses, and cells-only positive controls (see Additional file 1: Methods, for a detailed description of the assay procedure). The plates were incubated for 2 days at 37^o^ C; metabolic activity, serving as surrogate for live cell number, was assessed with the MTT assay (Sigma Aldrich), according to the manufacturer’s protocol.

### Statistical analysis

In the *S. cerevisiae* assays, each plate was scrutinized for outlier values by the Grubb’s test, implemented with the XLSTAT software package. When an outlier was identified, it was removed and replaced, as described in Additional file 1: Methods. Of note, no more than one outlier per column or row was removed. After reconciling outliers, the mean of the positive (cells-only) control wells was calculated and set to a value of 1; the plate data were normalized to this value.

The Combenefit software package (http://sourceforge.net/projects/combenefit/) calculates and displays the synergism-antagonism distributions and computes a variety of metrics from the distributions [45]. Although the software renders three models (Bliss, HAS, and Loewe), we chose the Loewe additivity model as the most suitable (see Discussion) because it allows for the possibility that the two drugs have interacting modes of action [49]. The Loewe model determines the degree of interaction by comparing the *experimental* concentration space to a *reference* concentration space. The latter is calculated from the individual dose-response curves for the two drugs in accordance with the assumptions of the Loewe model; thus, these must be included in each assay (Additional file 2: Fig. S1). The dose response curve for each of the chemotherapeutic drugs is computed by the software from all biologic replicates of that drug combination (see Figs. 2a,b; 3a,b; 4a,b; 5a,d; 7a,b,d,e,g,h,j,k). Dose response curves for lovastatin were also included in every assay; to maximize the reliability of the lovastatin dose-response curves, we pooled the lovastatin dose-response data from all ten dual drug sets (separately for days two and three). The mean dose response data so obtained for lovastatin were utilized in all ten experimental analyses.

After the Combenefit software compares the experimental surface to the reference surface, it assigns a synergy level to each cell in the matrix; statistical significance is then ascertained using the one-sample t-test [45]. If the test ascribes significance (indicated by: * *p* < 5 × 10–2; **p < 10–3, ***p < 10–4) to a given cell, the cell is colored appropriately to highlight its synergy or antagonism (see, for example, Fig. 2d).

## Results

### Evaluation of statin plus chemotherapeutic drug pairs in *S. cerevisiae*

#### Sorting of lovastatin/chemotherapeutic drug pairs using the global metric, “SUM_SYN_ANT”

**Fig. 1:**
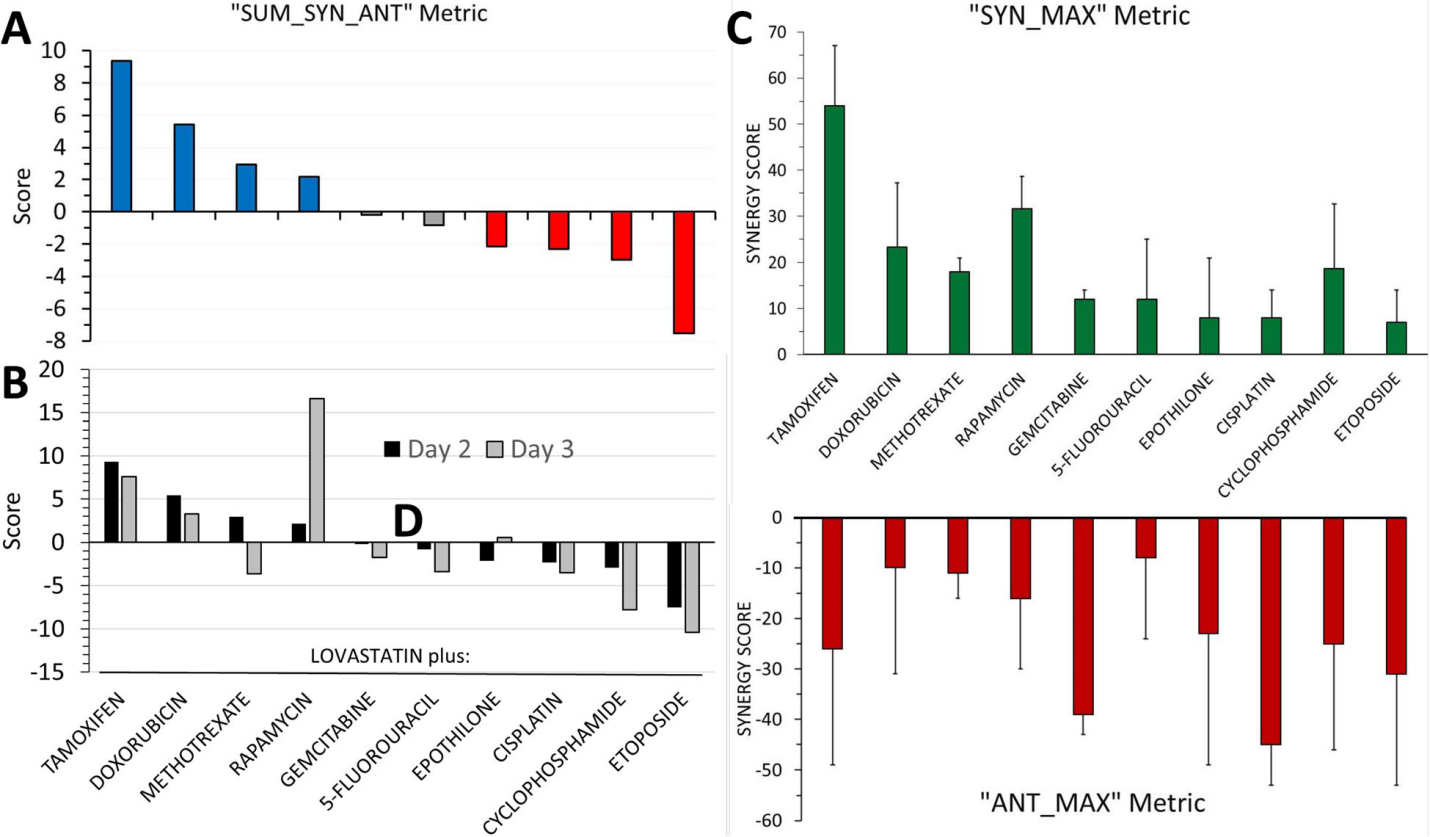
Graphical representation of three global metrics calculated by the Combenefit software for each of the ten dual-drug assays conducted with the *S. cerevisiae* heterozygous-deletion pool. **a**. The SUM_SYN_ANT metric, defined as the “sum of synergy and antagonism observed in concentration logarithmic space” [45], is depicted for the ten drug combinations on day two. The blue bars denote drug combinations with net synergism,; the red bars, net antagonism; and the grey bars, neutral scores. **b.** A comparison of SUM_SYN_ANT scores on Day 3 versus Day 2. In most, but not all instances (see text), the scores tended toward less synergism and/or greater antagonism on Day 3. **c.** The SYN_MAX metric (defined as the “maximum synergy observed”) on Day 2; the drug combinations are listed in the same order as in (**a**)**.** Standard deviations, as recorded by the software on the synergy level matrices for the Lowe model, are indicated. **d.** The ANT_MAX metric (“maximum antagonism observed”) and standard deviations on Day 2. The values in (**c**) and (**d**) represent *single* maximal values whereas the SUM_SYN_ANT metric encompasses *all* values within the concentration space

Most drug pairs demonstrated significant synergism and/or antagonism, depending upon the relative concentrations of the two drugs. Therefore, to broadly categorize the ten drug pairs, we exploited the global metrics generated by the *Combenfit* software. For this purpose, “SUM_SYN_ANT”, defined as the “sum of synergy and antagonism observed in concentration logarithmic space” ([45], Supplementary material), was the most useful. Applying this metric, drug pairs with a net score of > 2.0 were considered “synergistic” (four drug pairs), those with scores in the range of + 2.0 to − 2.0, as “neutral” (two drug pairs), and pairs with scores <− 2.0, as “antagonistic” (four drug pairs), Fig. 1a. With most drug combinations, the results obtained after 2 and 3 days of incubation were similar although the analyses on day three trended toward less synergism and/or greater antagonism, as illustrated by a comparison of the SUM_SYN_ANT scores calculated for the 2 days (Fig. 1b). The two noteworthy exceptions, methotrexate and rapamycin, are discussed below. When the data obtained on day three were congruent with the results on day two, only the latter are presented in this section; the day three data can otherwise be found in Additional file 2.

#### Chemotherapeutic drugs exhibiting net synergism with lovastatin

Based on the metric SUM_SYN_ANT on day two, and the criteria described above, tamoxifen (score, + 9.36) doxorubicin (+ 5.43), methotrexate (+ 2.93) and rapamycin (+ 2.16) each achieved net synergism when paired with lovastatin (Fig. 1a; Additional file 2, Table S1).

**Fig. 2:**
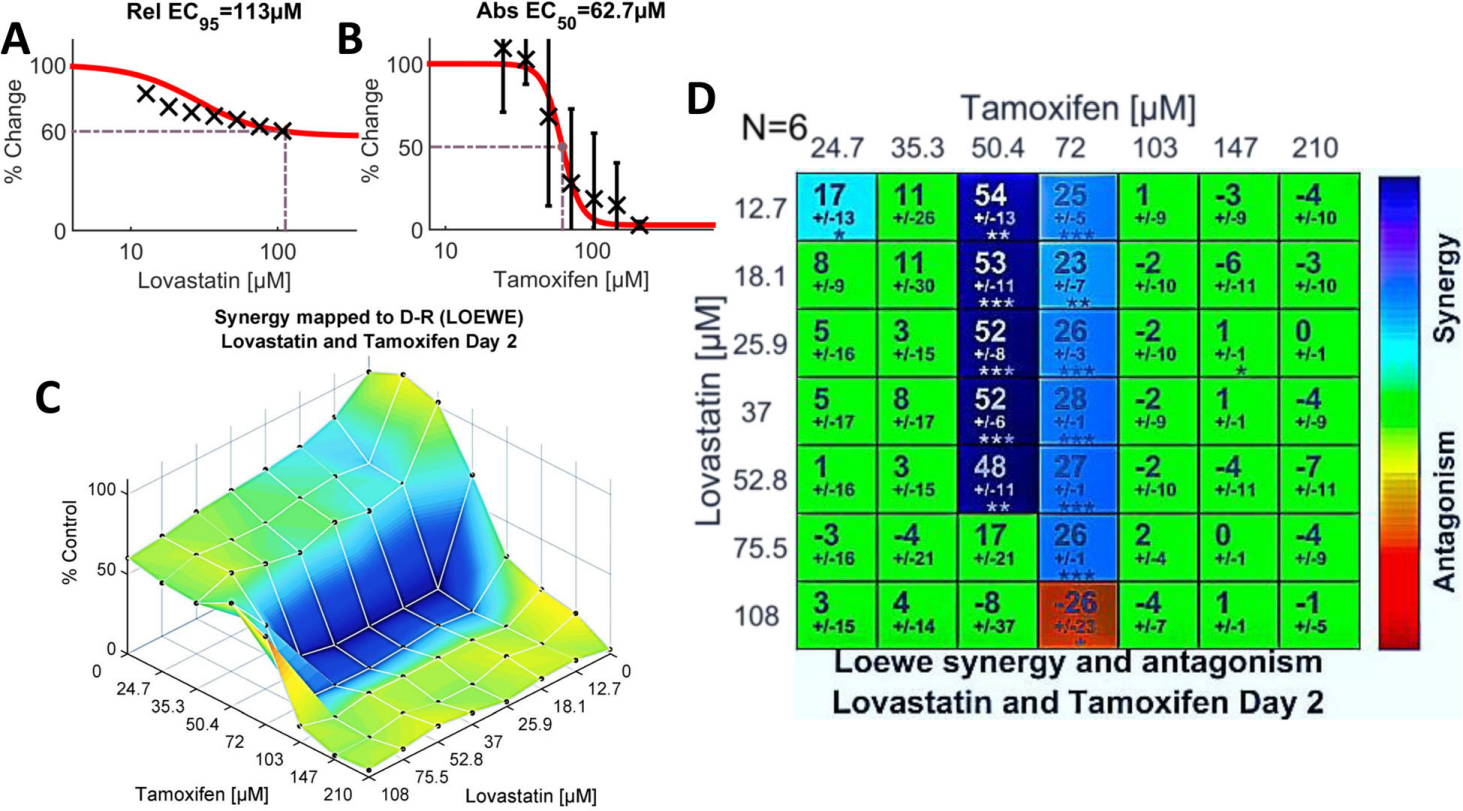
Strong synergism between tamoxifen and lovastatin evident from the dual-drug assays of tamoxifen plus lovastatin on Day 2. The data from six biologic replicates are included in the calculations. **a.** Single agent dose-response data and fitted curve for lovastatin, calculated from the normalized cell density data recorded from Column 9 of the 96-well plate (Additional file 2: Fig. S1). In the case of lovastatin, data from 49 experiments are aggregated to generate the curve (see Methods). **b.** Single agent dose-response data and fitted curve for tamoxifen (*N* = 6), generated from normalized Column 10 data (Additional file 2: Fig. S1). The Loewe model generates the reference concentration space from the two single agent dose-response curves. **c.** Shown here is the experimental two-drug combination dose-response surface, expressed as a percentage of the control value, calculated from the cells-only data and normalized to a value of 1 (see Methods). The plot thus portrays the efficacy of each of the 49 dual-drug combinations. Overlain on the dose-response surface are the Loewe synergy levels depicted in (d), colored without regard to statistical significance, according to the Synergism/Antagonism scale at the right of the graph. **d**. Shown in matrix format are synergy scores calculated according to the Loewe additivity model from the dual-drug experimental dose-response in comparison to the reference dose-response surface. The larger numeral in each box is the synergy score; negative values indicate antagonism. The number below the synergy score is the standard deviation. Boxes are coloured green if the synergy score is not significant. The boxes coloured according to the Synergism/Antagonism scale indicate results that are statistically significant by the one-sample t-test. The degrees of significance are as follows: * *p* < 5 × 10–2; **p < 10–3, ***p < 10–4. The number of biologic replicates (N) is indicated at the top left of the matrix

Tamoxifen, which earned the highest composite score (+ 9.36) on day two (Fig. 1a), also exhibited the greatest maximum synergy, + 53.6, (Fig. 1c and Fig. 2d). A score of + 53.6 represents about a 54% increase in effectiveness over that predicted if the two drugs were simply additive. The synergism between lovastatin and tamoxifen was evident over a broad range of concentrations of lovastatin but was narrowly confined to two concentrations of tamoxifen, 50.4 and 72 μM (Fig. 2d). Remarkably, a concentration of 72 μM of tamoxifen paired with 75.5 μM lovastatin resulted in a score of + 26 (*p* < .0001), yet a further 0.70 dilution of lovastatin (108 μM) at the same concentration of tamoxifen resulted in significant antagonism (score − 26, *p* < 0.05), Figs. 1d and 2d. The experimental combination dose response surface, with an overlay of synergy levels, is shown in Fig. 2c; this plot – a depiction of efficacy - illustrates the substantial inhibition of growth achieved within the synergistic space. Finally, we note that the synergy and antagonism matrix for tamoxifen plus lovastatin on day three (Additional file 2: Fig. S2d) shares a similar pattern with the day two results.

**Fig. 3:**
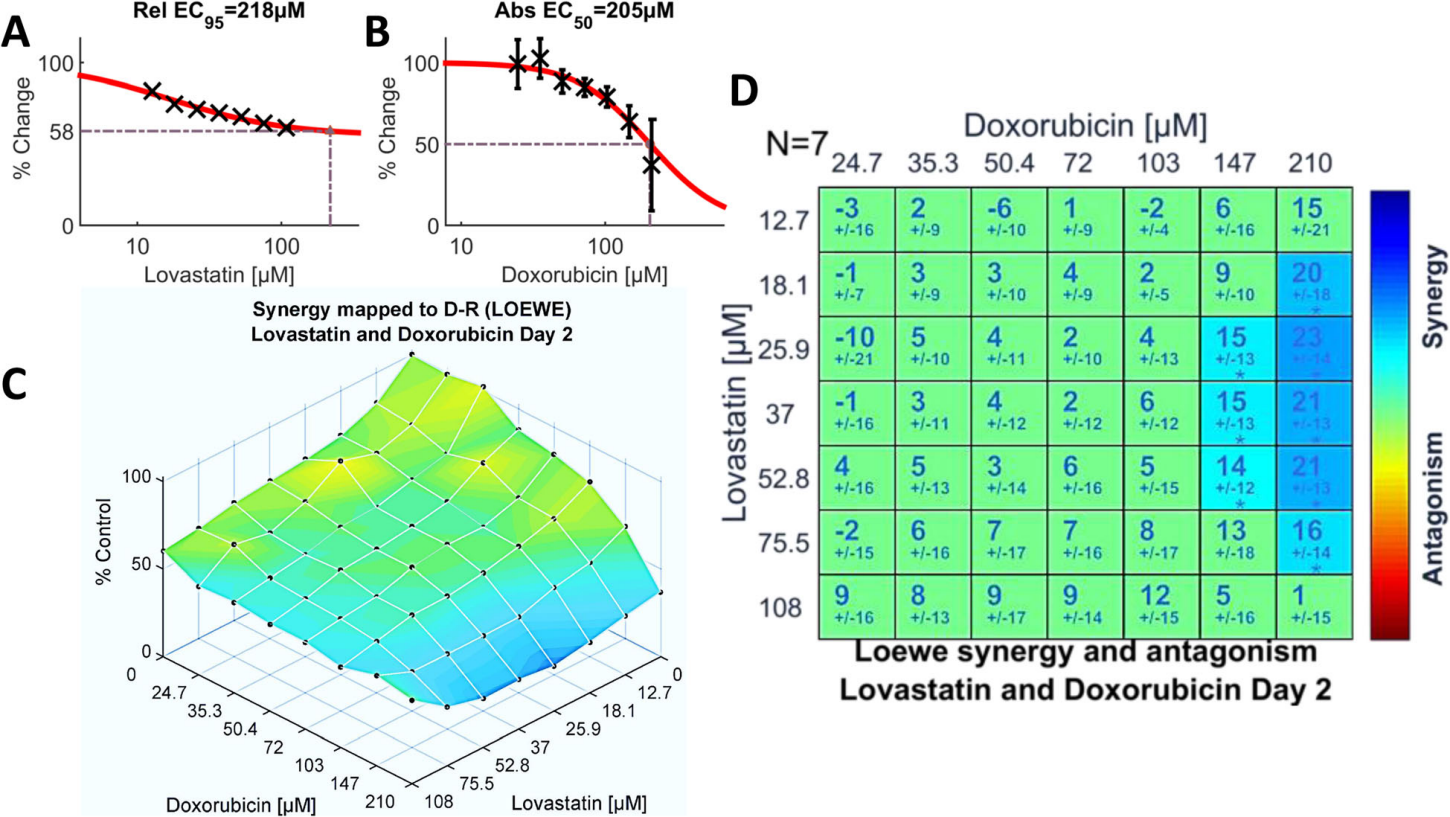
Dual drug assays of doxorubicin and lovastatin (*N* = 7) analyzed by the Loewe additivity model. **a.** and **b.** Single agent dose-response data and fitted curves compiled for lovastatin and doxorubicin, respectively. **c.** Efficacy of the dual-drug combinations overlain with synergy scores. **d.** Synergy levels calculated by the Loewe model and colored when significant. Only the highest two concentrations of doxorubicin exhibited statistically significant interactions with lovastatin. See the legend of Fig. 2 for details of the plots shown

The interactions of doxorubicin with lovastatin were statistically neutral over more than 80% of the concentration space (Fig. 3d); however, this combination earned the second highest composite score, + 5.43 (Fig. 1a; Additional file 2: Table S1) by virtue of the statistically significant synergism at the two highest concentrations of doxorubicin (147 and 210 μM) and the complete absence of significant antagonistic interactions (Fig. 1d and 3d). As was the case with tamoxifen, the significant synergistic concentrations of lovastatin embraced a wide range (18.1–75.5 μM) when paired with doxorubicin. Synergism was greatest on day 2 with a maximum score of 23.3 (Fig. 1c; Additional file 2: Table S1), trending downward on day 3 (Fig. 1b) but retaining a similar pattern (Additional file 2: Fig. S3a-d). Importantly, the efficacy (see Fig. 3c and Additional file 2: Fig. S3c) of the synergistic interactions was quite modest.

**Fig. 4:**
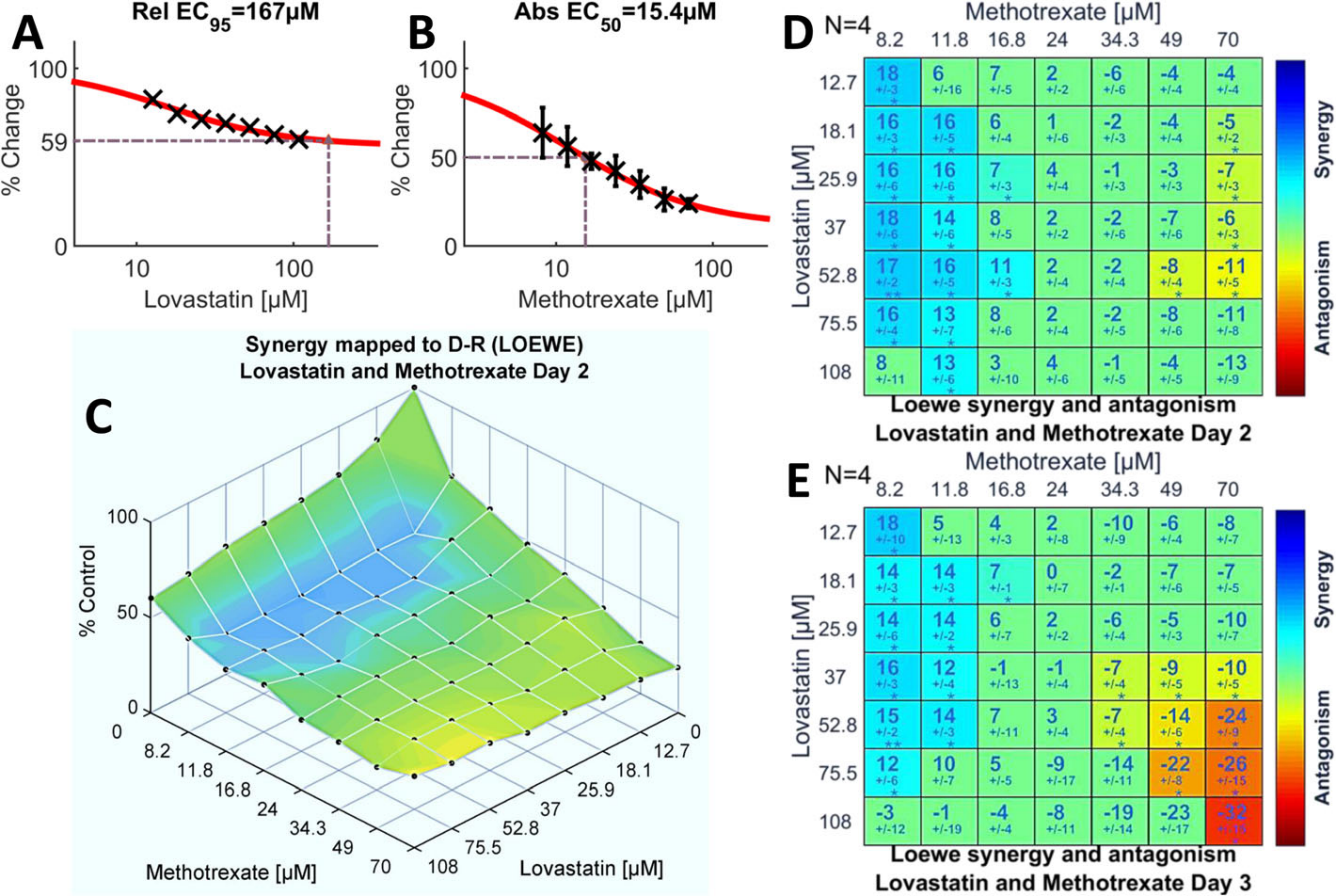
Lower concentrations of methotrexate synergize with the entire range of lovastatin concentrations tested. Shown are the single agent dose-response curves for lovastatin (**a**) and methotrexate (**b**). The experimental dose-response surface, (**c)**, illustrates the efficacy of the dual-drug combinations and is overlain with synergy and antagonism according to the Loewe model. **d**. The Loewe synergy level matrix on day two (*N* = 4) indicates the statistically significant synergy populating the concentration space framed by the lower concentrations of methotrexate and nearly the entire concentration range of lovastatin. Significant antagonism is detected only with the highest two concentrations of methotrexate. **e**. On day three (*N =* 4), the overall pattern of interactions is similar to that on day two but antagonism is more pronounced. A detailed description of the various plots is given in Fig. 2

The SUM_SYN_ANT score for the methotrexate-lovastatin pairing was a modest, perhaps misleading, + 2.93 (Fig. 1a): statistically significant synergism and antagonism coexisting in the concentration space (Figs. 1c, d and 4d) largely offset each other in the calculation of the global score. Importantly, the lower concentrations of methotrexate interacted synergistically with lovastatin while the higher methotrexate concentrations interacted antagonistically (Fig. 4d). The two lowest concentrations of methotrexate (8.2 and 11.8 μM) demonstrated statistically significant synergism over the entire concentration range of lovastatin, with maximum synergism (+ 17.9) evident at the lowest concentrations of both methotrexate and lovastatin (Fig. 4d). The antipodal concentrations of methotrexate (49 and 70 μM) demonstrated the greatest antagonism (− 13), Figs. 1d and 4d. This pattern persisted on day three but with lessened synergism and enhanced antagonism (Fig. 4e; Additional file 2: Fig. S4), causing the global score for methotrexate to flip from net synergism (+ 2.93) on day two to net antagonism (− 3.68) on day three (Fig. 1b).

**Fig. 5:**
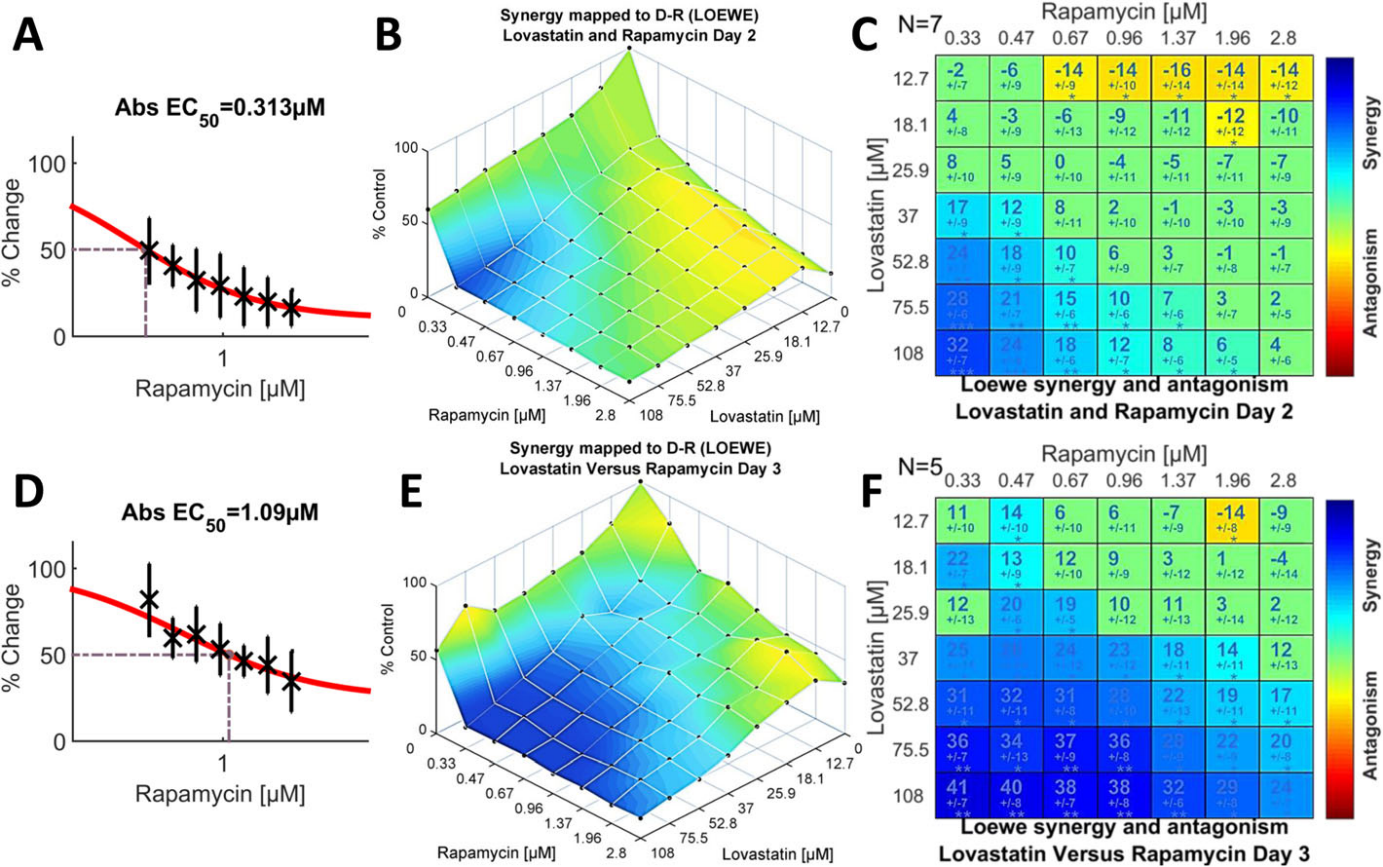
Drug interactions of rapamycin and lovastatin on days two (*N =* 7) and three (*N* = 5), indicating profound synergy between the two agents. The single agent dose-response curve for rapamycin is shown for day two (**a**) and day three (**d**). Note that the EC_50_ more than triples on day three. **b**. Rapamycin combined with lovastatin has substantial efficacy over much of the dose-response concentration space on day two. **e**. The dose-response space is similar on day three, despite the shift in the single agent dose-response curve for rapamycin (see **d**). The Loewe synergy level matrices on day two (**c**) and day three (**f**) are similar but with an even greater extent of statistically significant synergy at the later time point. The presence of strong antagonism, antipodal to the synergistic interactions, resulted in a modest global SUM_SYN_ANT score on day two (Fig. 1a) but the global metric reverses on day 3 (Fig. 1b) because of the expanding synergism and weakening antagonism (**f**)

Rapamycin, in combination with lovastatin, had a modest net score on day two (Fig. 1a) yet it achieved the highest synergism of all drug pairs on day three. In contrast to the trend of increasing antagonism from days 2 to 3 with other pairings, the SUM_SYN_ANT score for rapamycin on day three was nearly eight-fold that on day two (+ 16.6 versus + 2.16), Fig. 1b. As was the case with methotrexate, rapamycin paired with lovastatin exhibited both synergism and antagonism of statistical significance within its concentration spaces (Fig. 5c, f). On day 2, the synergistic and antagonistic interactions clustered toward opposing quadrants in the concentration space: lower concentrations of rapamycin paired with higher concentrations of lovastatin achieved significant synergism while higher concentrations of rapamycin and the two lowest concentrations of lovastatin were antagonistic (Fig. 5c). The maximum scores for synergism and antagonism were + 31.7 and − 15.6, respectively (Fig. 1c, d and Additional file 2: Table S1). Reflecting the coexistence of strong synergy and antagonism within the same concentration space, the SUM_SYN_ANT metric was a modest + 2.28. The overall pattern of rapamycin-lovastatin interactions persisted on day three, although synergism greatly increased, spreading further across the concentration space as antagonism receded (Fig. 5f); two-thirds of the cells recorded statistically significant synergism while only one cell retained significant antagonism. A comparison of efficacy afforded by the experimental dose-response space plots (Fig. 5b, e) suggests that the profound growth inhibition conferred by the synergistic interactions was similar on both days, an observation reinforced by comparing the contour maps of the dose response data (Additional file 2: Fig. S5). That the effects on cell growth were similar on days two and three, even though the absolute EC_50_ for rapamycin increased by about 3.5-fold on day three (Fig. 5d versus Fig. 5a), suggests that lovastatin somehow prolongs the efficacy of rapamycin.

#### Chemotherapeutic drug/lovastatin combinations registering neutral or antagonistic SUM_SYN_ANT scores

**Fig. 6:**
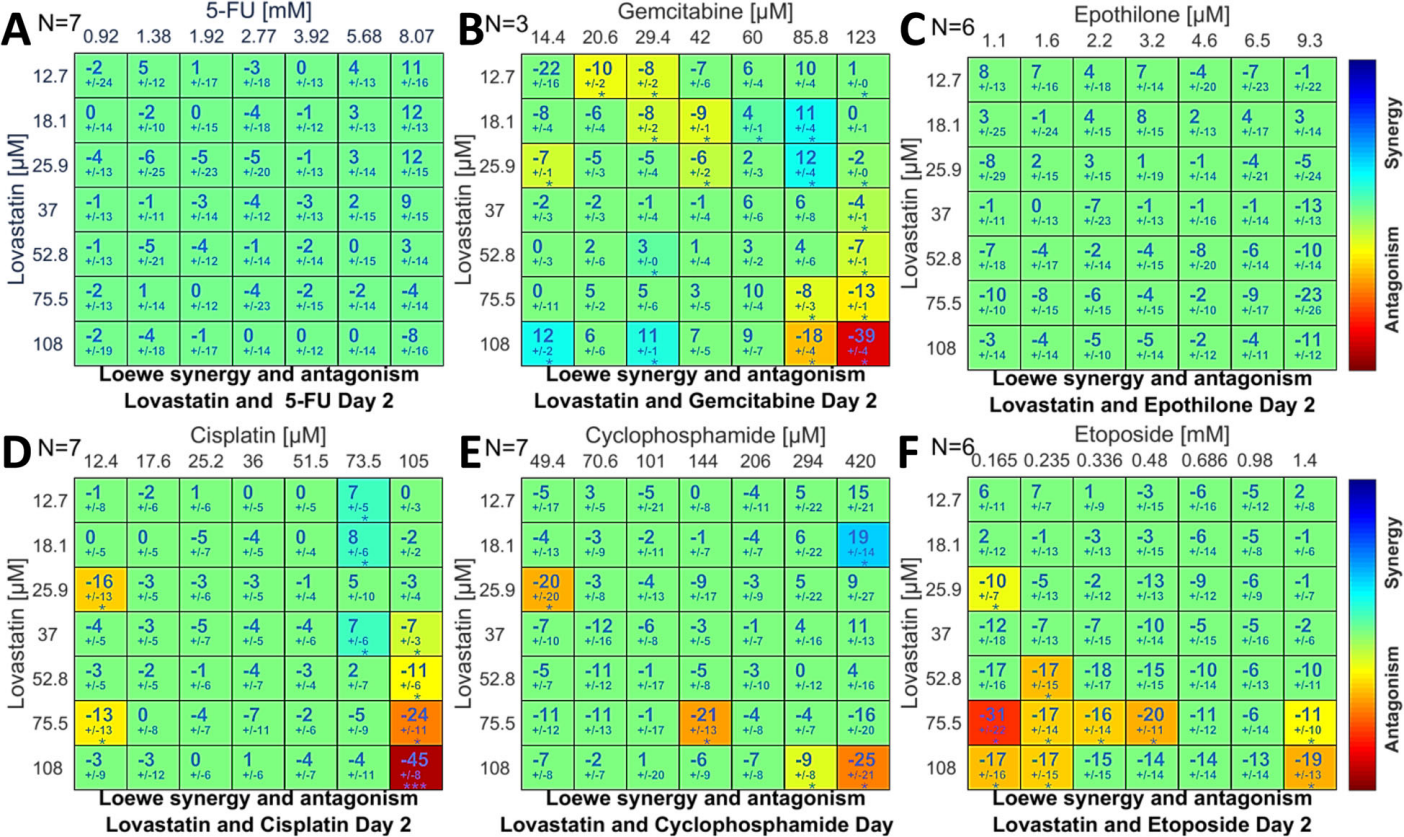
Loewe additivity matrices on day two of 5-fluorouracil (**a**), gemcitabine (**b**), epothilone (**c**), cisplatin (**d**), cyclophosphamide (**e**), and etoposide (**f**). The global metrics (Fig. 1a) were neutral or antagonistic for all six drugs in combination with lovastatin. The number of biologic replicates is specified in each panel

None of the six combinations with scores <+ 2 (Fig. 1a) exhibited strong synergism within the interaction matrices (Fig. 6a-f). Three patterns of synergy/antagonism were evident: 5-FU and epothilone had matrices totally devoid of significant interactions (Fig. 6a, c); gemcitabine, cisplatin and cyclophosphamide (Fig. 6b, d, e) registered a mixture of scattered, stronger antagonism interspersed with weaker synergism; and etoposide paired with lovastatin evoked only antagonism (Fig. 6f). The patterns were similar on day three (Additional file 2: Fig. S6a-f).

In sum, the interactions of lovastatin with the ten chemotherapeutic drugs proved to be selective: four drug pairs displayed net synergism yet for six pairs the interactions were either neutral or antagonistic.

### Evaluation of lovastatin plus tamoxifen in human cell lines

In order to validate the results generated by the yeast model, we submitted two of the drug combinations to further testing in human cell lines. Tamoxifen plus lovastatin was selected because this pairing produced the strongest net synergism on day two (Fig. 1a), even though yeast cells lack estrogen receptors, the principal target of tamoxifen in mammalian cells. Thus, the substantial effects of tamoxifen alone on growth (Fig. 2b), and its strong synergism with lovastatin (Fig. 2d), were quite unexpected (see Discussion). The second combination, lovastatin plus cisplatin, which serves as a surrogate for the antagonistic drug pairings (Fig. 1d), was selected because cisplatin is so widely prescribed for the treatment of a variety of human cancers, including lung, breast, liver, colon, and ovarian carcinomas [50].

#### Dual drug assays of tamoxifen and lovastatin in human breast and lung cancer cell lines

**Fig. 7:**
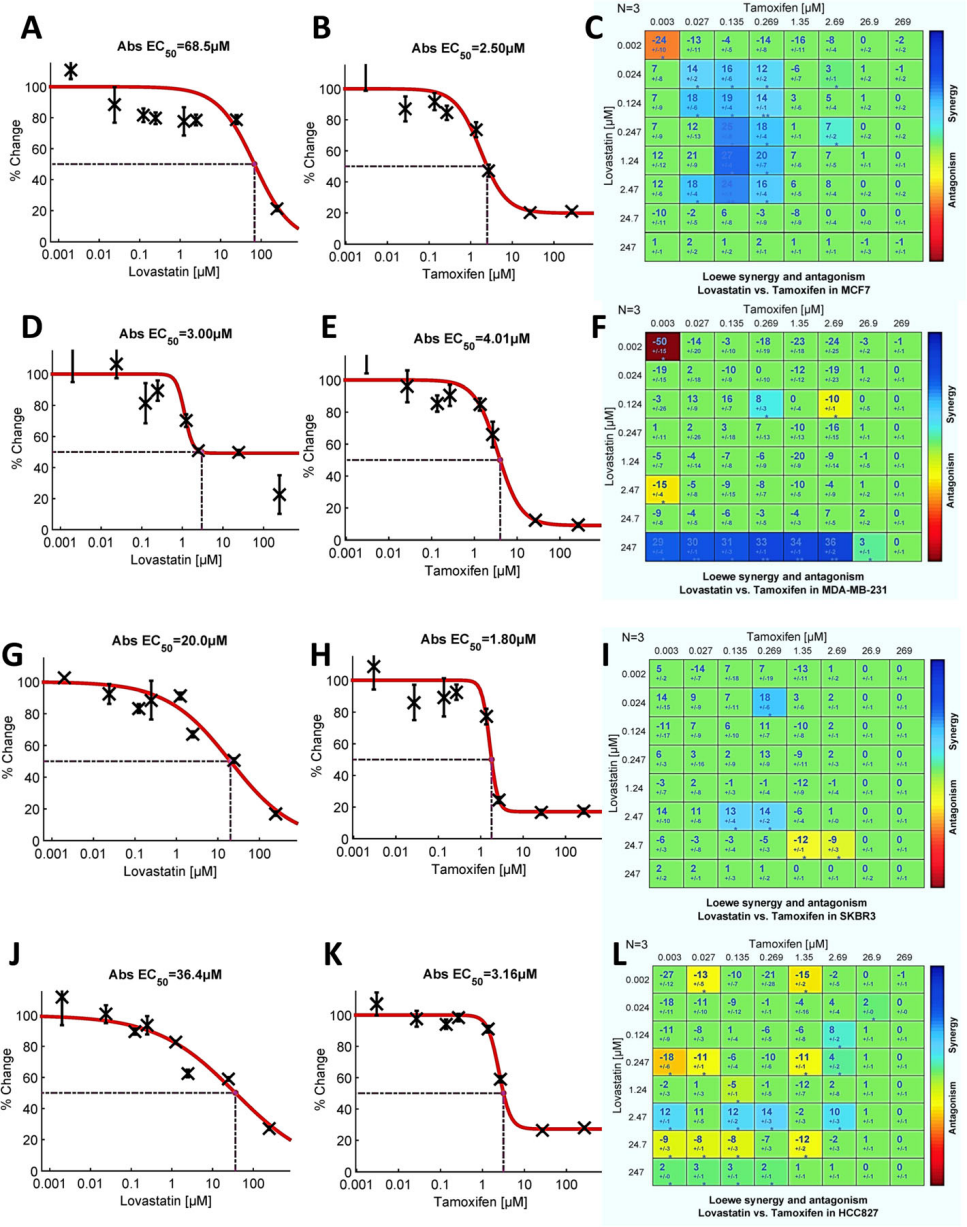
Tamoxifen and lovastatin dual-drug assays in human breast cancer cell lines, MCF7 (**a** – **c**), MDA-MB-231 (**d**- **f**), and SKBR3 (**g** – **i**), and lung adenocarcinoma (**j** – **l**). Shown are the single agent dose response data and the Loewe additivity model (synergy level) matrices. Note the variability of the lovastatin dose responses between cell lines (**a**, **d**, **g**, **j**). The tamoxifen single agent dose responses vary to a lesser degree (**b**, **e**, **h**, **k**); the EC_50_ values are clustered about the 1 μg/ml concentration. The Loewe synergy matrices (**c**, **f**, **i**, **l**), all of which include significant synergism and antagonism, are quite dissimilar. The concentration space of the estrogen receptor-positive MCF7 cell line (**c**) resembles that obtained with *S. cerevisiae* (see Fig. 2d). *N* = 3 for each cell line

The human breast cancer cell lines tested included one estrogen receptor (ER) positive, MCF7, and two ER negative, MDA-MB-231 and SK-BR-3 cell lines (see Methods). The results obtained with the ER positive MCF7 cell line are shown in Fig. 7a–c. The global metric SUM_SYN_ANT was + 93, the highest obtained in the cell line assays (note that the scale of dilutions differs from the assays in yeast). About 29% of the concentration space displayed synergism (Fig. 7c) with only one cell indicating statistically significant antagonism. Overall, the pattern is reminiscent of the assays of tamoxifen with lovastatin in *S. cerevisiae,* see Fig. 2d. The cell line MDA-MB-231 (Fig. 7d–f), from a triple negative breast carcinoma, exhibited both synergism and antagonism, resulting in a global metric of − 46.4. Although about twice as many cells displayed synergism as antagonism, strong antagonism (− 50.5) in one cell contributed substantially to the net score. The third breast cancer cell line, ER negative SK-BR-3 (Fig. 7g-i) displayed a similar pattern of scattered synergism and antagonism, generating a SUM_SYN_ANT score of + 24.0.

When tamoxifen and lovastatin were assayed in the lung adenocarcinoma cell line, HCC827 (Fig. 7j-l), the concentration landscape was weighted toward antagonism, as reflected in the SUM_SYN_ANT score of − 67.6. Scattered weak synergy was noted in about 10% of the concentration space.

#### Dual drug assays of cisplatin and lovastatin in human cancer cell lines

All three cell lines tested – A549, lung adenocarcinoma; HT-29, colon adenocarcinoma; and MCF7, breast carcinoma – exhibited negative (antagonistic) SUM_SYN_ANT scores, with weak maximum synergy and stronger maximum antagonism (Table 2; Additional file 2: Fig. S7a-f). The data from the three human cancer cell lines are entirely congruent with the comparable analysis in *S. cerevisiae* (Table 2).

**Table 2:** Global metrics of the drug combination lovastatin plus cisplatin in three cell lines: A549 (lung adenocarcinoma), HT29 (colon adenocarcinoma) and MCF7 (breast carcinoma), in comparison to the metrics obtained in similar *Saccharomyces cerevisiae* analyses

Metrics	*S. cerevisiae*		A549		HT29		MCF7	
	Day 2	Day 3	Day 2	Day 3	Day 2	Day 3	Day 2	Day 3
SUM_SYN_ANT	−2.335	−3.553	−7.642	−9.875	−5.887	− 3.117	−33.789	−13.498
SYN_MAX	13.895	15.698	6.014	4.812	15.346	11.252	12.558	4.922
ANT_MAX	−45.256	−35.016	−35.744	−43.200	−51.294	−55.074	−73.647	− 35.777

Together, the results with lovastatin in combination with cisplatin in three human cell lines and tamoxifen with MCF7 breast cancer cells are concordant with the findings in the *S. cerevisiae* assays. However, we note marked differences in the interactions of tamoxifen with lovastatin in ER negative breast cancer and lung cancer cell lines (see Discussion).

## Discussion

The fundamental question we sought to answer in this study is whether statin-chemotherapy interactions are a manifestation of a global effect of statins or are, as we hypothesized, specific to individual chemotherapeutic drugs. To that end, we assayed a single statin, lovastatin, paired with each of ten commonly prescribed chemotherapeutic agents, with various mechanisms of action (Table 1). The cellular substrate was the model organism, *Saccharomyces cerevisiae;* a balanced pool of heterozygous deletions of all essential genes was created for this study. Highly reproducible dilution assays were performed in microplates; cell densities were compiled with a plate reader. The data from the assays were submitted to rigorous statistical analysis to identify synergistic and antagonistic interactions, according to the Loewe additivity model, implemented with the Combenefit software package [45].

The Combenefit program compiles the data from the plate replicates for a given drug pair and computes various global metrics according to assumptions dictated by the chosen model. The metric “SUM_SYN_ANT”, which is as summation of all interactions within the concentration space, proved most useful. In accordance with our hypothesis, the ten chemotherapeutic agents exhibited a spectrum of global interactions, ranging from synergism to neutrality to antagonism (Fig. 1a). Four chemotherapeutic drugs paired with lovastatin exhibited strong, statistically significant, synergistic interactions: tamoxifen, doxorubicin, methotrexate and rapamycin. Two of the ten chemotherapeutic drugs - gemcitabine and 5-fluorouracil - had neutral interactions with lovastatin; and four – epothilone, cisplatin, cyclophosphamide and etoposide - exhibited net antagonism. Global metrics however provide only a snapshot of drug-drug interactions. The synergism score matrices were more informative; they revealed that synergism and antagonism often resided in the same concentration space, sometimes with potential clinical implications (see later).

Having demonstrated net synergism of four drug combinations in the yeast model, we selected one of them – tamoxifen and lovastatin - for additional study in human cancer cell lines. This combination, which exhibited the strongest synergy on day two, was intriguing because of its substantial efficacy in *S. cerevisiae,* which is devoid of estrogen receptors. (Although an estrogen binding protein has been identified in budding yeast [51, 52], the protein demonstrated negligible binding of tamoxifen [51].) The efficacy of tamoxifen alone (Fig. 2b) or paired with lovastatin (Fig. 2c, d) therefore likely results from activation of one or more of tamoxifen’s several known [47, 53–58], or as yet undiscovered, off-target pathways (reviewed in [53]).

Of the three human breast cancer cell lines assayed, MCF-7 – which possesses estrogen receptors – demonstrated strong synergy with lovastatin (Fig. 7c), in a pattern resembling that seen with yeast (Fig. 2d). In the other two breast cancer cell lines tested (see Fig. 7f, i), tamoxifen had only scattered, weak synergy or was strongly synergistic with only the highest concentration of lovastatin. Together, these observations frame a paradox: the combination of tamoxifen plus lovastatin was strongly synergistic in an organism, *S. cerevisiae*, devoid of estrogen receptors (ERs), yet displayed a similar pattern of strong synergism only in a breast cancer cell line, MCF7, which possesses ERs. (It is important when interpreting our experiments with tamoxifen in either yeast or the MCF7 cell line to remain cognizant of two well-documented observations: first, the parent drug tamoxifen – sometimes misleadingly labeled a “prodrug” [59, 60] – does *not* require its metabolism in order to be pharmacologically active [59–61]; and, second, tamoxifen has well-documented pharmacologic effects independent of the estrogen receptor [47, 53–58, 62].)

Curiously, the EC_50_ of lovastatin showed substantially greater variability between breast cancer cell lines than did the EC_50_ of tamoxifen. A possible resolution of these seeming contradictions is suggested by the reports of Radin and Patel [53] and Tan et al. [54]; higher concentrations (in the micromolar range) of tamoxifen are required to engage ER-independent pathways than for ER-dependent mechanisms. It therefore seems plausible that a statin might lend efficacy to tamoxifen in ER-negative breast cancer in which tamoxifen is otherwise impotent; however, targeted delivery of the two drugs would perhaps be required to achieve the requisite concentration ratios of the two drugs [63].

We sought further proof of principle in support of our *S. cerevisiae-*based experimental design by testing the combination of cisplatin and lovastatin in three human cell lines: A549 (lung adenocarcinoma); HT29 (colon adenocarcinoma); and MCF7 (breast carcinoma). This combination was chosen as a surrogate for the six neutral or antagonistic pairings because it so commonly prescribed for a variety of malignancies. In the yeast model, the combination exhibited strong antagonism (Table 2; Fig. 1a, d; Fig. 6d; Additional file 2: Fig. S6d). Congruent with our *S. cerevisiae* data, the metrics for the three cell lines were antagonistic, even more so than those calculated from the yeast assays (Table 2), lending further support to the validity of our model.

Two features of the experimental design merit further discussion. Because our intent was to compare the ten statin-chemotherapeutic drug pairs with each other, an important consideration was that the cellular target be a neutral one, free of the biologic and genetic biases inherent in every cancer cell line; choosing any one human cancer cell line for the assays risked biasing the assays for or against one or another drug pair. In the Background we set forth our rationale for choosing the model organism *Saccharomyces cerevisiae.* Although the utility of budding yeast in drug studies may be unfamiliar to some, there is ample precent for the choice. For example, three large chemogenomic studies [32, 34, 36] have been performed; these are consolidated in the searchable data base NetwoRx [33], which includes 466 drugs and compounds. (This data base, as well as other published literature, guided our selection of drug concentrations for the *S. cerevisiae* studies.) The balanced pool of heterozygous deletion strains of all essential genes, which we created in order to have a cellular substrate with consistent genetic diversity, proved to be only modestly more sensitive to the various drugs than the cognate wildtype strain. However, because each deletion is bar-coded, the pool provides a useful resource for the analysis of genetic targets and resistance mechanisms of drug treatments, studies beyond the scope of this report.

A second important consideration in experimental design was the choice of synergy model. The Combenefit software package [45] renders three models (Bliss, HSA and Loewe). The Bliss and Loewe model are arguably the most popular synergy models, but all synergy models have inherent flaws [49, 64, 65]. The probabilistic Bliss model assumes independent but competing drug actions whereas the Loewe additivity model assumes nonindependence; that is, the two drugs may interact with the same targets or pathways [49]. Because of the remarkable pleiotropy of statins (reviewed in detail in [66]), including interactions with a variety of signaling pathways, we posited nonindependence of lovastatin and the individual chemotherapeutic drugs and therefore chose the Loewe model as more appropriate.

Undue reliance upon global metrics, as for example in high throughput studies, risks overlooking potentially useful synergistic interactions which are confined to portions of the concentration space, often with coexisting antagonism (see Figs. 4d,e and 5c, f). Lehar et al. [65] posit that such patterns of dose-response surfaces of drug combinations are the consequence of drug-induced interacting pathways, both “on-target” and “off-target” [67, 68]. Lovastatin in combination with rapamycin proffers a case in point: lower concentrations of rapamycin interact synergistically with higher concentrations of lovastatin yet the converse yields significant antagonism (Fig. 5c, f). A comparison of the interactions of doxorubicin versus methotrexate with lovastatin further illustrate pitfalls of global metrics. The SUM_SYN_ANT score for doxorubicin is higher (+ 5.43) than for methotrexate (+ 2.93). However, the synergistic interactions for doxorubicin occur only with the highest two concentrations tested (Fig. 3d), a clinically problematic pattern. By contrast, the synergistic interactions of methotrexate are found with lower concentrations of both drugs (Fig. 4d, e). This suggests that adding a statin might increase the efficacy of methotrexate while allowing a reduction in its dosage, with attendant mitigation of methotrexate’s substantial toxicity. These observations lead to a sobering conclusion: the therapeutic consequences – be they advantageous or detrimental – of a given statin/chemotherapeutic drug combination may hinge upon the concentrations of each achieved at the tumor site. Consequently, targeted delivery strategies [63] with precise control of concentration ratios of the two drugs [69] may merit consideration.

In considering the Loewe synergy scores for the various drug interactions, we concur with the view “that any synergy model should be treated as an exploratory ranking statistic for prioritization of the most potent combinations for further evaluation…” [49]. To that end, we identified four chemotherapeutic drugs – tamoxifen, doxorubicin, methotrexate and rapamycin – which show strong synergistic interactions with lovastatin and merit further investigation. However, we stress that the data presented in this report should not be taken as - nor was our experimental approach intended to generate - prescriptive guidance for the clinical application of statins as adjuvants to conventional chemotherapeutic agents. That said, our results do identify statin/chemotherapeutic drug combinations warranting further study in cell lines, co-cultures, organoids and animal experiments.

In the Background we stated that an objective of our study was to illuminate the confounding literature bearing on the adjunctive role of statins in chemotherapy and cited the systemic reviews and meta-analyses by Farooqui et al. [24] and Mei et al. [23]. Farooqui et al. demonstrated that the addition of a statin to conventional therapy failed to improve progression-free or overall survival. Of the ten studies included in their analysis, six incorporated - either as the sole chemotherapeutic drug or as a component of a multi-drug regimen - agents which we found to be either neutral or antagonistic: etoposide, cisplatin, gemcitabine, and 5-FU (one protocol included both cisplatin and epirubicin, which is related to doxorubicin). Of the remaining four studies, one specified whole brain irradiation, and three incorporated drugs (afatinib, thalidomide and gefitinib) which we have not assayed. Thus, our data and the Farooqi meta-analysis provide mutually supportive, albeit circumstantial, evidence affirming the lack of efficacy of at least four specific statin plus chemotherapeutic drug combinations. Unfortunately, the larger systemic review by Mei et al. [23], which demonstrated beneficial effects of statins upon survival in cancer patients, did not specify the individual chemotherapeutic drugs used in the 95 studies included in their analysis.

## Conclusions

The data presented herein are potentially relevant to two clinical contexts when considering chemotherapeutic protocols. The first scenario is the purposeful inclusion of a statin in a chemotherapeutic drug regimen. If combinations of a statin with any of the four agents achieving synergy in our study – tamoxifen, doxorubicin, methotrexate and rapamycin – weather further scrutiny in in vitro cell line assays, co-culture or organoid studies, and in animal models, then – and only then - should clinical trials be considered.

The second clinical scenario is less obvious but no less important. Patients undergoing chemotherapy may also be receiving a statin, adventitiously co-prescribed for the treatment of elevated lipids or the prevention of cardiovascular disease. In the literature, numerous adverse interactions of various statins with many prescribed or over-the-counter drugs, herbs and other compounds have been documented (see, for example, [70–73]). In this context, the agents displaying antagonistic interactions with lovastatin - gemcitabine, epothilone, cisplatin, cyclophosphamide, and etoposide - generate especial concern. Absent further clinical studies, it would seem prudent for health care providers to weigh the perceived cardiovascular benefits of statins against the possible risks of decreased chemotherapeutic efficacy.

## Supplementary Information

**Additional file 1.**

**Additional file 2: Legends Figure S1.** Schematic diagram of the 96-well plate for the dual-drug assays (see Methods). The dose-responses (Columns 9 and 10), which are required for the computation of Loewe additive synergy, are performed at the same starting concentrations and with the same dilutions as in the dual-dilution portion of the plate. The format shown is compatible with the Combenefit software. BL **=** Plate blank (synthetic complete media + dextrose only). **Figure S2.** Tamoxifen plus lovastatin data on day 3. The comparable data for day two are depicted and described in Figure 2. The synergy matrix shown here is very similar to the day two matrix. **Figure S3.** Doxorubicin and lovastatin interactions on day three. The data are quite similar to the results on day two, shown in Figure 3. **Figure S4.** Methotrexate and lovastatin on day three. The patterns are quite similar to those recorded on day two (Fig. 4). The Loewe synergy matrix (D) is the same plot as shown in Fig. 4e. **Figure S5.** Experimental dose-response data for the combination of rapamycin and lovastatin. The combination dose-response data are visualized as contours, with 25, 50, 75 and 100% of control levels. Even though the EC50 of rapamycin is three-fold greater on day3 (Fig. 5, d vs. a), the 25% dose-response contour is achievable at all concentrations of rapamycin with only a modest increase in lovastatin concentration compared to day two. These data, supportive of the increase in synergy seen in the Loewe matrix (Fig. 5f compared to c), suggest that lovastatin somehow prolongs the efficacy of rapamycin. **Figure S6.** Day three Loewe synergy matrices corresponding to the day two data shown in Fig. 6. Epothilone plus lovastatin exhibits significant synergy on day three (C) whereas the combination did not on day two (Fig. 6c). The matrix for 5-fluoruracil (A) shows no significant interactions, as it did on day two. The other four drugs, gemcitabine (B), cisplatin (D), cyclophosphamide (E), and etoposide (F), exhibit greater antagonism on the third day. **Figure S7.** Loewe synergy matrices on days two and three for three cell lines in dual-drug assays of cisplatin in combination with lovastatin. Two human cancer cells lines, A549 (lung adenocarcinoma) (A, D) and MCF7 (breast cancer) (C, F), exhibited significant antagonism (and no significant synergism) on day two; the antagonism was more pronounced on day three. The HT29 colon adenocarcinoma cell line exhibited synergism and antagonism, both statistically significant, on day two (B) and to a lesser degree, on day three (E). The data shown here are comparable to that obtained with the *S. cerevisiae* assays (see Table 2, and Figs. 6c and S6D).

## Data Availability

The datasets generated and analyzed during the current study are available from the corresponding author on reasonable request. The relevant analyses are included in the published article [and its supplementary information files].

## Abbreviations

ER: Estrogen receptor
SCD: Synthetic complete medium with dextrose
SUM_SYN_ANT: Sum of synergy and antagonism observed in concentration logarithmic space

## Footnotes

1 The earliest known cases of cancer were discovered in Egyptian skeletons dating from circa 1200–2200 BCE. The antiquity of cancer is discussed in Binder, et al. [1].

## References

[1] Binder M, Roberts C, Spencer N, Antoine D, Cartwright C (2014). On the antiquity of cancer: evidence for metastatic carcinoma in a young man from ancient Nubia (c. 1200 BC). PLoS One.

[2] World Health Organization Releases Latest Global Cancer Data [https://www.cancerhealth.com/article/world-health-organization-releases-latest-global-cancer-data].

[3] Endo A (2010). A historical perspective on the discovery of statins. Proc Jpn Acad Ser B Phys Biol Sci.

[4] Endo A, Kuroda M (1976). Citrinin, an inhibitor of cholesterol synthesis. J Antibiot (Tokyo).

[5] Endo A, Kuroda M, Tanzawa K (1976). Competitive inhibition of 3-hydroxy-3-methylglutaryl coenzyme a reductase by ML-236A and ML-236B fungal metabolites, having hypocholesterolemic activity. FEBS Lett.

[6] Endo A, Kuroda M, Tsujita Y (1976). ML-236A, ML-236B, and ML-236C, new inhibitors of cholesterogenesis produced by Penicillium citrinium. J Antibiot (Tokyo).

[7] Brown AG, Smale TC, King TJ, Hasenkamp R, Thompson RH. Crystal and molecular structure of compactin, a new antifungal metabolite from Penicillium brevicompactum. J Chem Soc, Perkin Trans 1. 1976;(11):1165–70. 945291

[8] Oliver MF (1991). Might treatment of hypercholesterolaemia increase non-cardiac mortality?. Lancet (London, England).

[9] Newman TB, Hulley SB (1996). Carcinogenicity of lipid-lowering drugs. Jama.

[10] Demierre MF, Higgins PD, Gruber SB, Hawk E, Lippman SM (2005). Statins and cancer prevention. Nat Rev Cancer.

[11] Beckwitt CH, Brufsky A, Oltvai ZN, Wells A (2018). Statin drugs to reduce breast cancer recurrence and mortality. Breast Cancer Res.

[12] Hachem C, Morgan R, Johnson M, Kuebeler M, El-Serag H (2009). Statins and the risk of colorectal carcinoma: a nested case-control study in veterans with diabetes. Am J Gastroenterol.

[13] Huang WY, Li CH, Lin CL, Liang JA (2016). Long-term statin use in patients with lung cancer and dyslipidemia reduces the risk of death. Oncotarget.

[14] Singh S, Singh PP, Singh AG, Murad MH, Sanchez W (2013). Statins are associated with a reduced risk of hepatocellular cancer: a systematic review and meta-analysis. Gastroenterology.

[15] Zhang Y, Liang M, Sun C, Qu G, Shi T, Min M, Wu Y, Sun Y (2019). Statin use and risk of pancreatic Cancer: an updated meta-analysis of 26 studies. Pancreas.

[16] Raval AD, Thakker D, Negi H, Vyas A, Kaur H, Salkini MW (2016). Association between statins and clinical outcomes among men with prostate cancer: a systematic review and meta-analysis. Prostate Cancer Prostatic Dis.

[17] Iannelli F, Lombardi R, Milone MR, Pucci B, De Rienzo S, Budillon A, Bruzzese F (2018). Targeting Mevalonate pathway in Cancer treatment: repurposing of statins. Recent Pat Anticancer Drug Discov.

[18] Ding X, Zhang W, Li S, Yang H (2019). The role of cholesterol metabolism in cancer. Am J Cancer Res.

[19] Berger NA (2014). Obesity and cancer pathogenesis. Ann N Y Acad Sci.

[20] Matar P, Rozados VR, Roggero EA, Scharovsky OG (1998). Lovastatin inhibits tumor growth and metastasis development of a rat fibrosarcoma. Cancer Biother Radiopharm.

[21] Vallianou NG, Kostantinou A, Kougias M, Kazazis C (2014). Statins and cancer. Anti Cancer Agents Med Chem.

[22] Chae YK, Yousaf M, Malecek MK, Carneiro B, Chandra S, Kaplan J, Kalyan A, Sassano A, Platanias LC, Giles F (2015). Statins as anti-cancer therapy; can we translate preclinical and epidemiologic data into clinical benefit?. Discov Med.

[23] Mei Z, Liang M, Li L, Zhang Y, Wang Q, Yang W (2017). Effects of statins on cancer mortality and progression: a systematic review and meta-analysis of 95 cohorts including 1,111,407 individuals. Int J Cancer.

[24] Farooqi MAM, Malhotra N, Mukherjee SD, Sanger S, Dhesy-Thind SK, Ellis P, Leong DP (2018). Statin therapy in the treatment of active cancer: a systematic review and meta-analysis of randomized controlled trials. PLoS One.

[25] Nakamura CE, Abeles RH (1985). Mode of interaction of beta-hydroxy-beta-methylglutaryl coenzyme a reductase with strong binding inhibitors: compactin and related compounds. Biochemistry.

[26] Botstein D, Fink GR (2011). Yeast: an experimental organism for 21st century biology. Genetics.

[27] Botstein D, Chervitz SA, Cherry JM (1997). Yeast as a model organism. Science (New York, NY).

[28] Guaragnella N, Palermo V, Galli A, Moro L, Mazzoni C, Giannattasio S (2014). The expanding role of yeast in cancer research and diagnosis: insights into the function of the oncosuppressors p53 and BRCA1/2. FEMS Yeast Res.

[29] Skrzypek MS, Nash RS, Wong ED, MacPherson KA, Hellerstedt ST, Engel SR, Karra K, Weng S, Sheppard TK, Binkley G (2018). Saccharomyces genome database informs human biology. Nucleic Acids Res.

[30] Cabral ME, Figueroa LI, Fariña JI (2013). Synergistic antifungal activity of statin-azole associations as witnessed by Saccharomyces cerevisiae- and Candida utilis-bioassays and ergosterol quantification. Rev Iberoam Micol.

[31] Coorey NV, Sampson LD, Barber JM, Bellows DS (2014). Chemical genetic and chemogenomic analysis in yeast. Methods Mol Biol.

[32] Ericson E, Gebbia M, Heisler LE, Wildenhain J, Tyers M, Giaever G, Nislow C (2008). Off-target effects of psychoactive drugs revealed by genome-wide assays in yeast. PLoS Genet.

[33] Fortney K, Xie W, Kotlyar M, Griesman J, Kotseruba Y, Jurisica I (2013). NetwoRx: connecting drugs to networks and phenotypes in Saccharomyces cerevisiae. Nucleic Acids Res.

[34] Hillenmeyer ME, Fung E, Wildenhain J, Pierce SE, Hoon S, Lee W, Proctor M, St Onge RP, Tyers M, Koller D (2008). The chemical genomic portrait of yeast: uncovering a phenotype for all genes. Science (New York, NY).

[35] Hughes TR (2002). Yeast and drug discovery. Funct Integr Genomics.

[36] Parsons AB, Lopez A, Givoni IE, Williams DE, Gray CA, Porter J, Chua G, Sopko R, Brost RL, Ho CH (2006). Exploring the mode-of-action of bioactive compounds by chemical-genetic profiling in yeast. Cell.

[37] Reid RJ, Benedetti P, Bjornsti MA (1998). Yeast as a model organism for studying the actions of DNA topoisomerase-targeted drugs. Biochim Biophys Acta.

[38] Sturgeon CM, Kemmer D, Anderson HJ, Roberge M (2006). Yeast as a tool to uncover the cellular targets of drugs. Biotechnol J.

[39] Torres NP, Lee AY, Giaever G, Nislow C, Brown GW (2013). A high-throughput yeast assay identifies synergistic drug combinations. Assay Drug Dev Technol.

[40] Smith AM, Ammar R, Nislow C, Giaever G (2010). A survey of yeast genomic assays for drug and target discovery. Pharmacol Ther.

[41] Menacho-Marquez M, Murguia JR (2007). Yeast on drugs: Saccharomyces cerevisiae as a tool for anticancer drug research. Clin Transl Oncol.

[42] Giaever G, Nislow C (2014). The yeast deletion collection: a decade of functional genomics. Genetics.

[43] Lee W, St Onge RP, Proctor M, Flaherty P, Jordan MI, Arkin AP, Davis RW, Nislow C, Giaever G (2005). Genome-wide requirements for resistance to functionally distinct DNA-damaging agents. PLoS Genet.

[44] Wu HI, Brown JA, Dorie MJ, Lazzeroni L, Brown JM (2004). Genome-wide identification of genes conferring resistance to the anticancer agents cisplatin, oxaliplatin, and mitomycin C. Cancer Res.

[45] Di Veroli GY, Fornari C, Wang D, Mollard S, Bramhall JL, Richards FM, Jodrell DI (2016). Combenefit: an interactive platform for the analysis and visualization of drug combinations. Bioinformatics (Oxford, England).

[46] Loewe S (1953). The problem of synergism and antagonism of combined drugs. Arzneimittelforschung.

[47] Wiseman H, Cannon M, Arnstein HR (1989). Observation and significance of growth inhibition of Saccharomyces cerevisiae (A224A) by the anti-oestrogen drug tamoxifen. Biochem Soc Trans.

[48] Lorenz RT, Parks LW (1990). Effects of lovastatin (mevinolin) on sterol levels and on activity of azoles in Saccharomyces cerevisiae. Antimicrob Agents Chemother.

[49] Tang J, Wennerberg K, Aittokallio T (2015). What is synergy? The Saariselka agreement revisited. Front Pharmacol.

[50] Dasari S, Bernard Tchounwou P (2014). Cisplatin in cancer therapy: molecular mechanisms of action. Eur J Pharmacol.

[51] Burshell A, Stathis PA, Do Y, Miller SC, Feldman D (1984). Characterization of an estrogen-binding protein in the yeast Saccharomyces cerevisiae. J Biol Chem.

[52] Feldman D, Do Y, Burshell A, Stathis P, Loose DS (1982). An estrogen-binding protein and endogenous ligand in *Saccharomyces cerevisiae*: possible hormone receptor system. Science (New York, NY).

[53] Radin DP, Patel P (2016). Delineating the molecular mechanisms of tamoxifen's oncolytic actions in estrogen receptor-negative cancers. Eur J Pharmacol.

[54] Tan CK, Chow PK, Findlay M, Wong C, Machin D (2000). Use of tamoxifen in hepatocellular carcinoma: a review and paradigm shift. J Gastroenterol Hepatol.

[55] Wiseman H (1994). Tamoxifen and estrogens as membrane antioxidants: comparison with cholesterol. Methods Enzymol.

[56] Wiseman H, Cannon M, Arnstein HR (1990). Tamoxifen inhibits RNA and protein synthesis simultaneously in Saccharomyces cerevisiae: partial protection by antioxidants. Biochem Soc Trans.

[57] Wiseman H, Cannon M, Arnstein HR, Barlow DJ (1992). The structural mimicry of membrane sterols by tamoxifen: evidence from cholesterol coefficients and molecular-modelling for its action as a membrane anti-oxidant and an anti-cancer agent. Biochim Biophys Acta.

[58] Wiseman H, Laughton MJ, Arnstein HR, Cannon M, Halliwell B (1990). The antioxidant action of tamoxifen and its metabolites. Inhibition of lipid peroxidation. FEBS Lett.

[59] Lim YC, Li L, Desta Z, Zhao Q, Rae JM, Flockhart DA, Skaar TC (2006). Endoxifen, a secondary metabolite of tamoxifen, and 4-OH-tamoxifen induce similar changes in global gene expression patterns in MCF-7 breast cancer cells. J Pharmacol Exp Ther.

[60] Maximov PY, McDaniel RE, Fernandes DJ, Korostyshevskiy VR, Bhatta P, Mürdter TE, Flockhart DA, Jordan VC (2014). Simulation with cells in vitro of tamoxifen treatment in premenopausal breast cancer patients with different CYP2D6 genotypes. Br J Pharmacol.

[61] Brauch H, Mürdter TE, Eichelbaum M, Schwab M (2009). Pharmacogenomics of tamoxifen therapy. Clin Chem.

[62] Zheng A, Kallio A, Härkönen P (2007). Tamoxifen-induced rapid death of MCF-7 breast cancer cells is mediated via extracellularly signal-regulated kinase signaling and can be abrogated by estrogen. Endocrinology.

[63] Piccolo MT, Menale C, Crispi S (2015). Combined anticancer therapies: an overview of the latest applications. Anti Cancer Agents Med Chem.

[64] Vlot AHC, Aniceto N, Menden MP, Ulrich-Merzenich G, Bender A (2019). Applying synergy metrics to combination screening data: agreements, disagreements and pitfalls. Drug Discov Today.

[65] Lehar J, Zimmermann GR, Krueger AS, Molnar RA, Ledell JT, Heilbut AM, Short GF, Giusti LC, Nolan GP, Magid OA (2007). Chemical combination effects predict connectivity in biological systems. Mol Syst Biol.

[66] Gazzerro P, Proto MC, Gangemi G, Malfitano AM, Ciaglia E, Pisanti S, Santoro A, Laezza C, Bifulco M (2012). Pharmacological actions of statins: a critical appraisal in the management of cancer. Pharmacol Rev.

[67] Amelio I, Lisitsa A, Knight RA, Melino G, Antonov AV (2017). Polypharmacology of approved anticancer drugs. Curr Drug Targets.

[68] Anighoro A, Bajorath J, Rastelli G (2014). Polypharmacology: challenges and opportunities in drug discovery. J Med Chem.

[69] Mayer LD, Janoff AS (2007). Optimizing combination chemotherapy by controlling drug ratios. Mol Interv.

[70] Horodinschi RN, Stanescu AMA, Bratu OG, Pantea Stoian A, Radavoi DG, Diaconu CC. Treatment with Statins in Elderly Patients. Medicina (Kaunas). 2019;55(11). 10.3390/medicina55110721 PMC6915405 31671689

[71] Palleria C, Roberti R, Iannone LF, Tallarico M, Barbieri MA, Vero A, Manti A, De Sarro G, Spina E, Russo E (2020). Clinically relevant drug interactions between statins and antidepressants. J Clin Pharm Ther.

[72] Siwek M, Woroń J, Gorostowicz A, Wordliczek J (2020). Adverse effects of interactions between antipsychotics and medications used in the treatment of cardiovascular disorders. Pharmacol Rep.

[73] Wang S, Li W, Yang J, Yang Z, Yang C, Jin H (2020). Research Progress of herbal medicines on drug metabolizing enzymes: consideration based on toxicology. Curr Drug Metab.

