# Supplementary material for: Complex interactions of lovastatin with 10 chemotherapeutic drugs: a rigorous evaluation of synergism and antagonism
Source: BMC Cancer. 2021 Apr 6;21:356. doi: 10.1186/s12885-021-07963-w (PMC8022429; doi:10.1186/s12885-021-07963-w)
Supplement: Supplementary file 1 — Additional file 1. [file 12885_2021_7963_MOESM1_ESM.pdf]

Additional File 1

***Saccharomyces cerevisiae* heterozygous deletion pool**

Each strain was plated to YPD plus G418 and incubated at 30º C for two to four days, then resuspended in YPD. The individual cell suspensions were diluted to a set concentration, pooled, concentrated by centrifugation, then aliquoted and frozen in 15% sterile glycerol.

***Saccharomyces cerevisiae* dose response curves**

Appropriate serial dilutions of individual drugs in SCD were performed in 96-well tissue culture plates; the final volume per well was 75 µl. Then, 50 µl of pool cells were added to each well. Each plate also included positive controls (75 µL of SCD plus 50 µL cells) and a plate blank or negative control (125 µl SCD). Plates were sealed and incubated at 29ºC with shaking (Jitterbug Shaker, Boekel Scientific, Feasterville, PA, USA). Cell densities (OD_{600}) were read after two and three days of incubation using the Biotek Synergy 4 plate reader (Biotek Instruments, Inc., Winooski, VT, USA). The data were normalized by setting the mean of the positive control readings to a value of 1. An appropriate concentration range - encompassing the convexity, inflection point, and concavity of the dose response curve - was then selected for each drug.

***Saccharomyces cerevisiae* dual drug dilution assay**

Well A1 – the plate blank – contains 125 µl SCD. The remainder of Column 1 (B1 – H1) is loaded with 250 µl of the initial concentration of the chemotherapeutic drug and wells A2 – A8 with 250 µl of the initial concentration of lovastatin. The remaining wells of the matrix (B2 –

H8), initially loaded with 75 µl SCD, are assigned to the cross-wise 7 X 7, 70% serial dilutions, performed as follows: 175 µl are removed from wells A2 – A8 and added to B2 – B8 and mixed by pipetting, followed by removal of 175 µl for addition to the next row down, *etc.* The process is then repeated beginning with wells B1 – H1 and continuing to Column 8. Columns 9 (A9 – H9) and 10 (A10 – H10) are reserved for dose response curves of lovastatin and the chemotherapeutic agent, respectively, again using identical 70% serial dilutions of the compounds. Wells A11 – H12 (which contain cells but no drug, thus serving as positive controls) are initially loaded with 75 µl SCD. Thus, the final volume in every well (except A1) before the addition of cells is 75 µl. Then, 50 µL of pooled cells are added to each well, omitting A1.

Assays in Human Cell Lines

The 96 well tissue culture plate was first seeded with $5*10^3$ cells in 100 µl of the appropriate medium (omitting only the plate blank well) and incubated overnight at 37° C. Then, 10 µl of media were withdrawn from each well designated for drug treatment and replaced with 5 µl each of appropriately pre-diluted lovastatin and tamoxifen solutions in accordance with the crosswise dilution format. The final concentrations of lovastatin and tamoxifen are denoted in the relevant illustrations.

Statistical Analysis

In the *S. cerevisiae* assays, it was noted that the OD_{600} density values in column 12 (“cells only”) exhibited inordinate variability compared to the cells-only positive controls in the adjacent column 11. We ascribed this to an “edge effect” related to the forced air temperature regulation mechanism of the shaker. Consequently, we discarded the column 12 values from every experiment. Each plate was then scrutinized for individual outlier values by the Grubb’s

test, implemented with the XLSTAT software package. This test identifies no more than one outlier per column or row. Because the Combenefit software requires that the submitted tabular dataset not have missing data, the values removed as outliers were replaced according to the following rules: (1) An outlier removed from the cells-only control column 11 is replaced with the mean of the remaining seven cells; (2) An outlier removed from a dose response curve (columns 9 and 10) is replaced with the mean of the values above and below it; and (3) an outlier removed from the crosswise dilution matrix is replaced by the mean of its two to four (depending on location) nearest neighbors. After removing and replacing outliers, the mean of column 11 (cells-only positive controls) was calculated and set to a value of 1; the remaining plate data were normalized to this value.

Because in the yeast assays the dose-response curve for lovastatin is gradual, diminishing by only about 20 per cent over the range of concentrations chosen, computation of its dose response curve is highly sensitive to even minor variations in the data from those wells (A9 - H9, see Figure 1). Therefore, for greater reliability we pooled the lovastatin dose-response data from all ten dual drug sets (49 assays) separately for days two and three. The mean dose response data so obtained for lovastatin were utilized in all ten experimental analyses (see, for example, Figure 3A).
