# Supplementary material for: Complex interactions of lovastatin with 10 chemotherapeutic drugs: a rigorous evaluation of synergism and antagonism
Source: BMC Cancer. 2021 Apr 6;21:356. doi: 10.1186/s12885-021-07963-w (PMC8022429; doi:10.1186/s12885-021-07963-w)
Supplement: Supplementary file 2 — Additional file 2: Legends Figure S1. Schematic diagram of the 96-well plate for the dual-drug assays (see Methods). The dose-responses (Columns 9 and 10), which are required for the computation of Loewe additive synergy, are performed at the same starting concentrations and with the same dilutions as in the dual-dilution portion of the plate. The format shown is compatible with the Combenefit software. BL = Plate blank (synthetic complete media + dextrose only). Figure S2. Tamoxifen plus lovastatin data on day 3. The comparable data for day two are depicted and described in Figure 2. The synergy matrix shown here is very similar to the day two matrix. Figure S3. Doxorubicin and lovastatin interactions on day three. The data are quite similar to the results on day two, shown in Figure 3. Figure S4. Methotrexate and lovastatin on day three. The patterns are quite similar to those recorded on day two (Fig. 4). The Loewe synergy matrix (D) is the same plot as shown in Fig. 4e. Figure S5. Experimental dose-response data for the combination of rapamycin and lovastatin. The combination dose-response data are visualized as contours, with 25, 50, 75 and 100% of control levels. Even though the EC50 of rapamycin is three-fold greater on day3 (Fig. 5, d vs. a), the 25% dose-response contour is achievable at all concentrations of rapamycin with only a modest increase in lovastatin concentration compared to day two. These data, supportive of the increase in synergy seen in the Loewe matrix (Fig. 5f compared to c), suggest that lovastatin somehow prolongs the efficacy of rapamycin. Figure S6. Day three Loewe synergy matrices corresponding to the day two data shown in Fig. 6. Epothilone plus lovastatin exhibits significant synergy on day three (C) whereas the combination did not on day two (Fig. 6c). The matrix for 5-fluoruracil (A) shows no significant interactions, as it did on day two. The other four drugs, gemcitabine (B), cisplatin (D), cyclophosphamide (E), and etoposide (F) [file 12885_2021_7963_MOESM2_ESM.zip › Additional File 2 correctedR2.pdf]

Additional File 2 Legends

Figure S1. Schematic diagram of the 96-well plate for the dual-drug assays (see Methods). The dose-responses (Columns 9 and 10), which are required for the computation of Loewe additive synergy, are performed at the same starting concentrations and with the same dilutions as in the dual-dilution portion of the plate. The format shown is compatible with the Combenefit software. **BL =** Plate blank (synthetic complete media + dextrose only).

Figure S2. Tamoxifen plus lovastatin data on day 3. The comparable data for day two are depicted and described in Figure 2. The synergy matrix shown here is very similar to the day two matrix.

Figure S3. Doxorubicin and lovastatin interactions on day three. The data are quite similar to the results on day two, shown in Figure 3.

Figure S4. Methotrexate and lovastatin on day three. The patterns are quite similar to those recorded on day two (Figure 4). The Loewe synergy matrix **(D)** is the same plot as shown in Figure 4E.

Figure S5. Experimental dose-response data for the combination of rapamycin and lovastatin. The combination dose-response data are visualized as contours, with 25%, 50%, 75% and 100% of control levels. Even though the EC_{50} of rapamycin is three-fold greater on day3 (Figure 5, D vs. A), the 25% dose-response contour is achievable at all concentrations of rapamycin with only a modest increase in lovastatin concentration compared to day two. These data, supportive of the increase in synergy seen in the Loewe matrix (Figure 5F compared to C), suggest that lovastatin somehow prolongs the efficacy of rapamycin.

Figure S6. Day three Loewe synergy matrices corresponding to the day two data shown in Figure 6. Epothilone plus lovastatin exhibits significant synergy on day three **(C)** whereas the combination did not on day two (Figure 6C). The matrix for 5-fluoruracil **(A)** shows no significant interactions, as it did on day two. The other four drugs, gemcitabine **(B)**, cisplatin **(D),** cyclophosphamide **(E)**, and etoposide **(F)**, exhibit greater antagonism on the third day.

Figure S7. Loewe synergy matrices on days two and three for three cell lines in dual-drug assays of cisplatin in combination with lovastatin. Two human cancer cells lines, A549 (lung adenocarcinoma) **(A,D)** and MCF7 (breast cancer) **(C,F)**, exhibited significant antagonism (and no significant synergism) on day two; the antagonism was more pronounced on day three. The HT29 colon adenocarcinoma cell line exhibited synergism and antagonism, both statistically significant, on day two **(B)** and to a lesser degree, on day three **(E)**. The data shown here are comparable to that obtained with the *S. cerevisiae* assays (see Table 2, and Figures 6C and S6D).
